# Kinase-Based Taming of Brain Microglia Toward Disease-Modifying Therapy

**DOI:** 10.3389/fncel.2018.00474

**Published:** 2018-12-05

**Authors:** Sun-Hwa Lee, Kyoungho Suk

**Affiliations:** ^1^New Drug Development Center, Daegu Gyeongbuk Medical Innovation Foundation, Daegu, South Korea; ^2^Department of Pharmacology, Brain Science and Engineering Institute, School of Medicine, Kyungpook National University, Daegu, South Korea

**Keywords:** microglia, neuroinflammation, protein kinase, drug target, neurodegenerative disease

## Abstract

Microglia are the primary immune cells residing in the central nervous system (CNS), where they play essential roles in the health and disease. Depending on the CNS inflammatory milieu, they exist in either resting or activated states. Chronic neuroinflammation mediated by activated microglia is now considered to be a common characteristic shared by many neurodegenerative diseases such as Parkinson’s disease, Alzheimer’s disease, and amyotrophic lateral sclerosis, which currently pose a significant socioeconomic burden to the global healthcare system. Accumulating evidence has indicated protein kinases (PKs) as important drug targets for therapeutic interventions of these detrimental diseases. Here, we review recent findings suggesting that selected PKs potentially participate in microglia-mediated neuroinflammation. Taming microglial phenotypes by modulating the activity of these PKs holds great promise for the development of disease-modifying therapies for many neurodegenerative diseases.

## Introduction

Human kinases primarily comprise phosphotransferase, which catalyzes the transfer of the γ-phosphoryl group of adenosine triphosphate (ATP) to the hydroxyl group of a tyrosine (Tyr), serine (Ser), threonine (Thr), or histidine (His) residue of proteins and other target molecules including lipids and sugars. The kinases are known to regulate a multitude of cellular signaling pathways such as cell proliferation, survival, growth, apoptosis, differentiation, cytoskeletal rearrangement, metabolism, and angiogenesis. Aberrant kinase activity is linked to a wide range of diseases including neoplastic diseases, central nervous system (CNS) disorders, vascular disorders, and chronic inflammatory diseases. Except for only one protein His kinase and a couple of dual-specificity protein kinases (PKs) capable of phosphorylating both Tyr and Ser/Thr residues on their target molecules, PKs broadly fall into two groups: the protein Tyr kinases (PTKs) and the Ser- and Thr-specific PKs (SPKs) (Rask-Andersen et al., 2014). While the SPKs constitute a majority of the PKs, the PTKs serve as important drug targets due to their strong association of gain and/or loss of function mutations with various diseases. It is noteworthy that most of the US FDA-approved kinase inhibitors are PTK inhibitors for cancer indications, but not for CNS indications (Gunosewoyo et al., 2017).

Microglia are primary immune cells residing in the CNS, where they play key roles in the development and function of a normal healthy brain. Under normal physiological conditions, ramified microglia constantly monitor the brain microenvironment in search of the presence of tissue damage and pathogen infections by continuously protruding and retracting their long processes. In addition to this surveillant function, microglia exert a phagocytic function to detect and rapidly eliminate degenerating neurons, resulting in the prevention of further detrimental effect on neighboring cells. This microglial phagocytic function is known to be crucial for synapse maturation (Paolicelli et al., 2011; Schafer et al., 2012; Gomez-Nicola and Perry, 2015). Moreover, microglia are directly or indirectly involved in the modulation of neuronal activity at the synapse, and influence myelination and neurogenesis by delivering signals in primary myelinating areas of the developing brain (Wlodarczyk et al., 2017). However, depending on encountered stimuli, microglia are activated and undergo significant changes in their function and morphology. Morphologically, they change to a rounded amoeboid form with shortened processes from a ramified form with long processes. Functionally, they result in either ‘classical’ pro-inflammatory or ‘alternative’ anti-inflammatory phenotypes (Arcuri et al., 2017; Kabba et al., 2018). Although neurons, oligodendrocytes, and astrocytes also play important roles in CNS inflammatory responses, it is currently viewed that chronic inflammation mediated by microglia predominantly contributes to many CNS disorders (Glass et al., 2010; Ransohoff, 2016b; Shabab et al., 2017).

**FIGURE 1:**
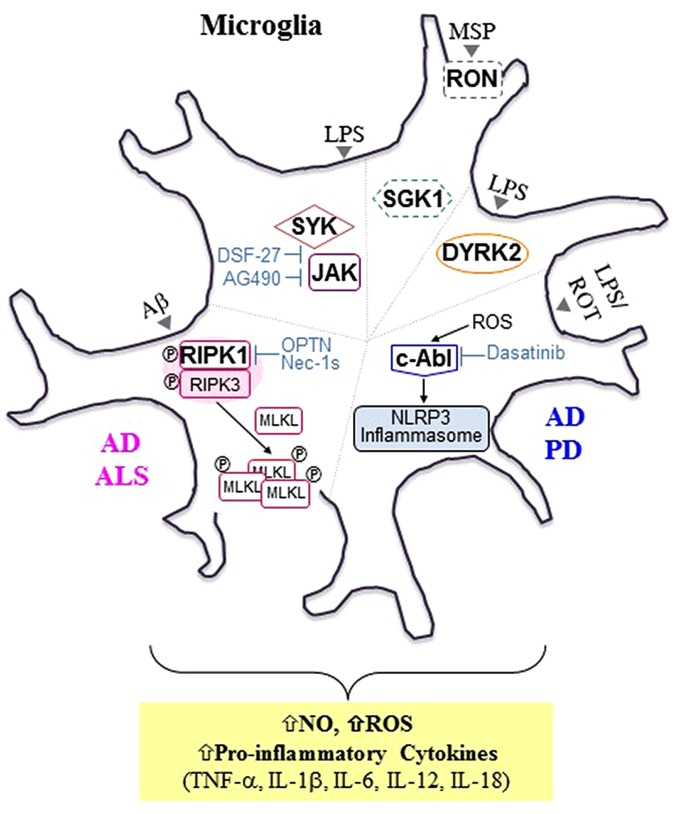
Selected microglial protein kinases involved in microglia-mediated neuroinflammation and neurodegenerative diseases. In addition to progressive neuronal damage and death, it is now evident that chronic neuroinflammation mediated by microglia is another common hallmark shared by neurodegenerative diseases such as Alzheimer’s disease (AD), Parkinson’s disease (PD), and amyotrophic lateral sclerosis (ALS). Selected protein kinases (PKs) involved in microglia-mediated neuroinflammation are presented here, and these PKs can be targeted to tame microglial phenotypes favorable for disease-modifying treatments. In general, activated receptor interacting protein kinase 1 (RIPK1), a serine (Ser)/threonine (Thr) PK, interacts with and phosphorylates RIPK3, forming a RIPK1/RIPK3-containing complex, which is known as a “ripoptosome.” Activated RIPK3, in turn, recruits and phosphorylates mixed lineage kinase domain-like protein (MLKL), promoting MLKL oligomerization and subsequent translocation into the plasma membrane, leading to direct pore formation and the initiation of necroptosis. In animal models of AD [APP/ PS1 transgenic mice] and ALS [optineurin (Optn) knockout mice (KO) and mSOD^G93A^ mice], activated RIPK1 (either by Aβ_1-42_ peptide or by the presence of Optn KO or mSOD^G93A^) mediates inflammatory responses rather than necroptosis in microglia (Ito et al., 2016; Ofengeim et al., 2017). In addition, axonal myelination defects were observed in ALS mouse models and elevated levels of hallmarks of necroptosis were detected in microglia in postmortem cortical samples from AD patients. Treatment with Nec-1s, a highly selective and central nervous system-permeable RIPK inhibitor, was demonstrated to inhibit microglia-mediated inflammation (Ito et al., 2016). Reactive oxygen species (ROS) derived from oxidative stress were found to activate nucleotide oligomerization domain (NOD)-like receptor protein (NLRP3) inflammasome via cellular Abelson murine leukemia viral oncogene homolog 1 (c-Abl) activation, resulting in the release of primarily interleukin (Iβ)-1b and IL-18 (Lawana et al., 2017). Treatment of microglia with dasatinib, a dual c- Abl/c-Src kinase inhibitor, was demonstrated to inhibit NLRP3-mediated release of pro-inflammatory factors including IL-1bβ, IL-18, ROS, nitric oxide (NO), and tumor necrosis factor-α (TNF-α). A strong correlation between inflammasomes and neurological disorders including AD, PD, and multiple sclerosis (MS) has been implicated in many recent studies (Lang et al., 2018). Both spleen tyrosine kinase (SYK) and Janus kinase (JAK), intracellular tyrosine (Tyr) PKs were also found to be associated with microglia-mediated neuroinflammation upon lipopolysaccharide (LPS) stimulation (Zeng et al., 2014). DSF-27, a novel sesquiterpene dimmer isolated from the medicinal plant, *Artemisia argyi*, was demonstrated to inhibit LPS-induced activation of SYK and JAK signaling pathways, resulting in a reduction in TNF-α, IL-1β, IL-6, and NO levels. Treatment with AG490, a specific inhibitor of JAK2, was found to inhibit LPS-induced NO production (Inoue et al., 2016; Asai et al., 2018). Dual-specificity tyrosine phosphorylation-related kinase 2 (DYRK2) is an intracellular Ser/Thr kinase localized in both the nucleus and the cytoplasm. It was demonstrated to translocate to the nucleus and interact with multiple kinases downstream of LPS stimulation, thereby regulating the production of pro-inflammatory molecules such as TNF-α and IL-1β. Unlike the other kinases presented here, the basal activity of both Receptor D’Origine Nantais (RON) and serum- and glucocorticoid-inducible kinase 1 (SGK1), boxed with the dotted line, were demonstrated to be involved in the anti-inflammatory process in microglia. RON, a receptor tyrosine kinase, is activated by its ligand, macrophage-stimulating protein (MSP), resulting in the promotion of anti-inflammatory responses and subsequent tissue repair (Dey et al., 2018). SGK1, an intracellular Ser/Thr kinase, was found to inhibit LPS-induced activation of microglia. Aβ, amyloid beta; ROT, rotenone; APP, Amyloid Precursor Protein; PS1, Presenilin 1; Nec-1s, necrostatin-1s.

Several recent review articles have shed light on the PKs as attractive drug targets for many diseases in the CNS. However, they primarily focused on PKs in neurons (Chico et al., 2009; Martin et al., 2013; Tell and Hilgeroth, 2013; Dzamko et al., 2014; Mehdi et al., 2016; Gunosewoyo et al., 2017), rather than those in microglia (Lee and Suk, 2017). Here, we briefly review recent findings on selected microglial PKs involved in microglia-mediated neuroinflammation (Figure 1), a common underlying mechanism of many neurodegenerative diseases including Parkinson’s disease (PD), Alzheimer’s disease (AD), and amyotrophic lateral sclerosis (ALS).

## Microglia-Mediated Neuroinflammation

The incidence of neurodegenerative diseases, including AD, PD, and ALS, is growing worldwide, thereby posing a considerable social and economic burden to the global healthcare system (Gitler et al., 2017). One of the common characteristics shared by these diseases is the loss of a specific population of neurons. AD is caused by the progressive loss of not only pyramidal neurons in the hippocampus and cortex, but cholinergic neurons in the basal forebrain. PD is caused by the progressive degeneration of dopamine neurons in the substantia nigra (SN) pars compacta of the midbrain as well as in other brain areas. ALS is characterized by the loss of motor neurons in the primary motor cortex, brainstem, and spinal cord (Ransohoff, 2016b; Lee and Suk, 2018). However, loss of neurons is not the only characteristic of neurodegenerative diseases. Accumulating evidence implies that neuronal degeneration may activate glial cells such as microglia, astrocytes, and oligodendrocytes, resulting in amplification of neuroinflammation. It is now widely accepted that microglia-mediated chronic inflammation is responsible for the progressive loss of neurons and serves as another hallmark common to neurodegenerative and neurodevelopmental diseases (Glass et al., 2010; Mosher and Wyss-Coray, 2014; Ransohoff, 2016b; Shabab et al., 2017). Furthermore, microglia-induced neurotoxicity as well as oxidative stress are also considered as other common pathological features shared by virtually all neurodegenerative diseases (Lee and Suk, 2018).

Microglial activation is often classified as either ‘classical’ or ‘alternative’ (Jha et al., 2016). ‘Alternative’ activation of microglia is induced by various cytokines [interleukin-4 (IL-4), IL-10, and IL-13], or immune complexes or apoptotic cells, resulting in the production of anti-inflammatory cytokines (IL-10 and transforming growth factor β), neurotrophic and growth factors (Jha et al., 2016; Gosselin et al., 2017). Thus, alternatively activated microglia refers to a beneficial and neuroprotective phenotype, functioning to resolve inflammation, to promote brain repair, and to eliminate cellular debris. In contrast, ‘classical’ activation of microglia is induced by lipopolysaccharide (LPS), ATP, interferon (IFN)-γ, and granulocyte-macrophage colony-stimulating factor, resulting in the production of pro-inflammatory cytokines [tumor necrosis factor-α (TNF-α), IL-1β, IL-6, and IL-18] and chemokines. In addition, they secrete oxidative stress-related molecules [nitric oxide (NO), reactive oxygen species (ROS), and superoxide anions]. Thus, classically activated microglia often refers to a neurotoxic and destructive phenotype, aggravating neuroinflammation and brain damage (Jha et al., 2016; Lee and Suk, 2018).

**FIGURE 2:**
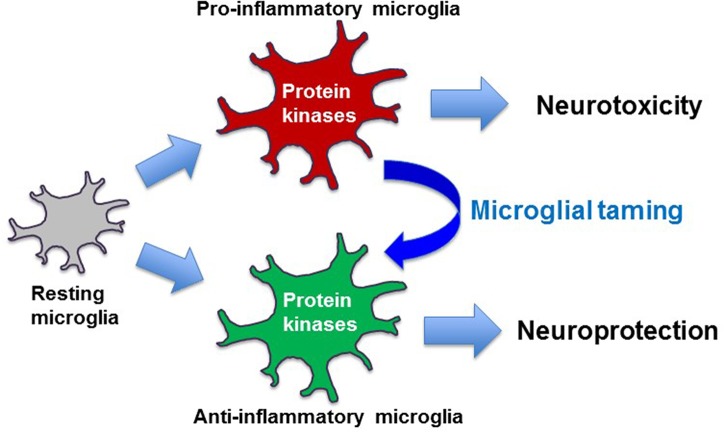
Taming of microglia toward disease-modifying therapy. Pro-inflammatory microglia exert neurotoxic effects, while anti-inflammatory microglia are often neurotrophic and neuroprotective. Protein kinase-based modulation of microglial phenotypes from pro-inflammatory to anti-inflammatory may form the basis of disease-modifying therapy.

While the contributing roles of these two microglial phenotypes in neuroinflammation have been widely presented, microglia exhibit a high degree of heterogeneity (Jha et al., 2016). Depending on the brain area and the disease stages, both microglial phenotypes simultaneously exist in the same pathological brain. Moreover, the presence of more complex and distinct phenotypes of microglia has recently been reported (Bisht et al., 2016; Keren-Shaul et al., 2017; Krasemann et al., 2017). Indeed, *in vivo* activation status of microglia is more likely a continuum of these two phenotypes. Thus, simple classification of microglial activation as either classical or alternative, often referred to as M1 or M2, respectively, does not adequately reflect the complexity of microglial activation. Nonetheless, therapeutic strategies targeting neuroinflammation mediated by microglia are currently focused on the development of glial phenotype modulators (GPMs) that promote the M2 phenotype while suppressing the M1 phenotype of activated microglia (Ransohoff, 2016a; Song and Suk, 2017; Suk, 2017; Lee and Suk, 2018) (Figure 2).

## Protein Kinases in Microglia-Mediated Neuroinflammation

For the last two decades, PKs have been intensively pursued by both pharmaceutical industries and academia as attractive drug targets for not only cancer, but also inflammatory, infectious, degenerative, metabolic and cardiovascular diseases. However, most of kinase inhibitors have been approved for cancer indications rather than CNS indications (Gunosewoyo et al., 2017). Nonetheless, an increasing number of PKs have emerged as potential drug targets for CNS disorders (Chico et al., 2009; Gunosewoyo et al., 2017; Lee and Suk, 2017). Thus, this review summarizes selected PKs that are potentially involved in microglial activation and concomitant induction of neuroinflammation (Figure 1).

### Receptor Interacting Protein Kinases

The receptor interacting protein kinase (RIPK) family contains seven members: RIPK1, RIPK2, RIPK3, RIPK4, RIPK5, RIPK6, and RIPK7. All members of the RIPK family contain an N-terminal Ser/Thr kinase domain (KD) (Ekdahl, 2012). Besides N-terminal KD, RIPK1 has a death domain (DD) and a bridging intermediate domain (ID) harboring an RIP homotypic interaction motif (RHIM) at the C-terminus, while RIPK2 has a caspase activation and recruitment domain (CARD) and ID without an RHIM at the C-terminus. RIPK3 contains a unique C-terminal sequences harboring an RHIM but lacks an ID. Both RIPK4 and RIPK5 contain ankyrin domains and an ID at the C-terminus. Both RIPK6 and RIPK7, which are structurally distant from the other family members, have several unique domains such as leucine-rich repeat (LRR) regions. Thus, RIPK6 and RIPK7 also refer to LRR kinase 1 (LRRK1) and 2 (LRRK2), respectively (Humphries et al., 2015). While there is currently a limited understanding of the physiological functions of RIPK4 – RIPK7, numerous studies have reported the important molecular and physiological roles of RIPK1 – RIPK3 in inflammation and cell death (Ślusarczyk et al., 2018). RIPK1 is now recognized as a master regulator of necroptosis, a regulated cell death characterized by loss of cell membrane integrity, swelling of cytoplasm and organelle, lack of DNA fragmentation, mitochondrial dysfunction and cellular collapse, resulting in deleterious signaling pathways downstream of type 1 TNF- α receptor (TNFR1). Upon TNF-α stimulation, caspase-8 activation is known to induce apoptosis. However, when caspase-8 dependent apoptosis is defective, RIPK1 is recruited to the intracellular DD of TNFR1 and interacts with RIPK3, inducing phosphorylation of RIPK3 and formation of an RIPK1/RIPK3-containing complex, which is known as complex IIb (Cho et al., 2009; He et al., 2009). RIPK3, then, initiates necroptosis by recruiting and phosphorylating mixed lineage kinase domain-like protein (MLKL), which is oligomerized and inserted into the plasma membrane to form pores (Figure 1) (Sun et al., 2012; Cai and Liu, 2014; Wang et al., 2014).

Recent studies have demonstrated that microglial RIPK1-mediated necroptosis is closely associated with the pathogenesis of AD and ALS. Ofengeim et al. (2017) reported for the first time that inhibition of RIPK1 kinase activity is effective in alleviating inflammation mediated by microglia *in vitro* and in mouse models of AD. This study demonstrated that elevated levels of RIPK1 were detected in microglia in postmortem cortical samples from AD patients as well as in amyloid precursor protein (APP)/presenilin 1 (PS1) transgenic (Tg) mouse model of AD. In addition, the levels of phosphorylation of RIPK1 on the Ser166 residue, a marker for RIPK1 autophosphorylation and activation, was found to be elevated in primary microglia treated with the Aβ_1-42_ peptide. Either treatment of Nec-1s, a selective and CNS-permeable RIPK1 inhibitor, or by introduction of a kinase dead mutation of RIPK1 (RIPK1^D138N/D138N^) was shown to reduce pro-inflammatory cytokines (TNF-α and IL-6) in primary microglia in response to the Aβ_1-42_ peptide as well as in the CNS of APP/PS1 Tg mice. Interestingly, memory deficits of APP/PS1 Tg mice was significantly improved by administration of Nec-1s or by genetic inhibition of RIPK1. By analyzing the transcriptional profiles of APP/PS1 microglia, this study revealed that RIPK1 in primary microglia is involved in the upregulation of Cst7 and Ch25h, which were previously identified in adult microglia obtained from animal models of AD (the 5xFAD Tg mice) and ALS (the mSOD1^G93A^ Tg mice), as well as aged mice. Ch25h encodes an enzyme on the cell surface responsible for cholesterol and lipid metabolism, and was previously demonstrated as a point of focus in genome-wide association studies of AD (Wollmer, 2010). Cst7 encodes Cystatin F, an endosomal/lysosomal cathepsin inhibitor, and was recently identified as one of the markers for disease-associated microglia (DAM), which is a newly characterized microglial phenotype found in the diseased brain (Papassotiropoulos et al., 2005; Keren-Shaul et al., 2017). This RIPK1-mediated induction of Cst7 was demonstrated to impair lysosomal trafficking of microglia, resulting in reduced clearing of Aβ. Administration of Nec-1s lowered Cst7 expression levels in APP/PS Tg mice (Ofengeim et al., 2017).

Ito et al. (2016) recently demonstrated that microglial RIPK1 can provoke toxicity of motor neuron axons by using optineurin (Optn) knockout (KO) mice as a model of ALS. The functional loss of OPTN has been associated with both familial and sporadic cases of ALS (Beeldman et al., 2015). The authors found that the Optn N KO mice exhibited enhanced levels of multiple necroptotic markers [RIPK1, RIPK3, phosphorylation of MLKL (p-MLKL), and the complex IIb] both in the spinal cords and in the mouse embryo fibroblasts. Interestingly, it was demonstrated that Optn KO microglia exhibited increased p-RIPK1 on Ser14 and Ser15 residues, indicators of activated RIPK1, compared to that in wild-type (WT) microglia, and that this increased level of p-RIPK1 was inhibited by Nec-1s treatment and by RIPK1^D138N/D138N^. In addition, the Optn KO mice showed elevated levels of several pro-inflammatory cytokines in the spinal cords, but not in those of the Optn KO mice carrying RIPK1^D138N/D138N^. Since microglia express little MLKL, but increased p-RIPK1 levels, the authors suggested that RIPK1 promotes pro-inflammatory signaling, rather than cell death. By performing RNA sequencing on primary microglia obtained from WT, Optn KO mice, and Optn KO mice carrying RIPK1^D138N/D138N^, this study revealed that elevated levels of apoptosis, CD14 and CD86, indicators of the pro-inflammatory state, and axonal myelination defects found in Optn KO mice were not shown in Optn KO mice carrying RIPK1^D138N/D138N^ or by administration of Nec-1s. These results were further confirmed in mSOD^G93A^ Tg mice. Lastly, this study demonstrated that necroptotic markers (RIPK1, MLKL, p-RIPK1, pMLKL, and RIPK3) were also elevated in both microglia and oligodendrocytes in human ALS samples. Collectively, this study suggests that microglial RIPK1 plays a crucial role in inducting inflammatory signaling pathways in ALS.

Given that the level of RIPK1 expression is higher than that of RIPK3 in activated microglia, RIPK1 inhibition may be an effective approach to attenuate microglia-mediated inflammatory responses. In addition, considering that RIPK1 is involved in signaling pathways downstream of TNFR1, rather than TNFR2, inhibiting RIPK1 may provide a novel approach to selectively target the detrimental cellular activities mediated by TNFR1 signaling pathways (Dhib-Jalbut and Kalvakolanu, 2015).

### Cellular Abelson Murine Leukemia Viral Oncogene Homolog 1

Cellular abelson murine leukemia viral oncogene homolog 1 (c-Abl) (ABL1) is a member of the Abl family, which also comprises the Abl-related gene (Are or ABL2) as a member. c-Abl is known to be activated by diverse stimuli including oxidative stress, DNA damage, growth factors, and cell adhesion, thereby affecting various cellular activities including cell growth, survival, motility, proliferation, cytoskeleton reorganization, DNA repair, receptor endocytosis, autophagy, and oxidative stress (Gonfloni et al., 2012; Lindholm et al., 2016). Earlier studies have provided evidence that c-Abl plays multiple roles in neuronal development by positively regulating synapse formation, neurulation, and dendrogenesis (Schlatterer et al., 2011), and that aberrant activation of c-Abl is shown to be associated with AD and PD (Derkinderen et al., 2005; Ko et al., 2010; Tremblay et al., 2010; Imam et al., 2011; Gonfloni et al., 2012; Lindholm et al., 2016). Indeed, accumulating evidence has implied the beneficial effect of c-Abl inhibition for patients with AD and PD (Schlatterer et al., 2011; Karuppagounder et al., 2014; Mahul-Mellier et al., 2014; Brahmachari et al., 2016). Nilotinib, an FDA-approved Abl inhibitor for patients with chronic myeloid leukemia, are currently undergoing phase II clinical trials for both AD and PD (Lee and Suk, 2017). Nonetheless, there are limited studies regarding the function of c-Abl in the microglia-mediated neuroinflammatory process in AD and PD (Deleidi et al., 2010; Field et al., 2010; Dhawan and Combs, 2012; Cheng et al., 2017; Lee and Suk, 2017).

Inflammasomes are multiprotein complexes assembled in response to damage signals and invading pathogens. They are presently categorized into two families: the protein family containing pyrin and hematopoietic-interferon-inducible nuclear antigens with 200 amino-acid repeats domain and the nucleotide oligomerization domain (NOD)-like receptor protein (NLR) protein family phylogenetically containing three subfamilies: NODs, IPAF, and NLRPs. Among 14 members of NLPRs, the most well characterized inflammasome is NLRP3 inflammasome consisting of NLRP3, the apoptosis associated speck-like protein containing a CARD domain, and pro-caspase-1 (Rubartelli, 2014; Lang et al., 2018). The NLRP3 inflammasome is tightly controlled and induced by two steps. The first step is the transcriptional induction of pro-IL-1β and NLRP3 by activation of nuclear factor kappa-light-chain-enhancer of activated B cells (NF-κB) via Toll-like receptors (TLRs) or NOD2 signaling. The second step is the formation of the NLRP3 inflammasome complex and subsequent production of mature IL-1β and IL-18 processed by caspase-1 (Ozaki et al., 2015).

Lawana et al. (2017) recently reported that c-Abl is involved in microglial activation of the NLRP3 inflammasome in a BV-2 immortalized murine microglial cell line (Bocchini et al., 1992). This study demonstrated that compared to BV-2 cells treated with either LPS or rotenone (ROT), a mitochondrial electron transport chain inhibitor, LPS-primed BV-2 cells treated with ROT (LPS/ROT) exhibited a significant increase in NLRP3 inflammasome markers (NLRP3, mature caspase-1, and IL-1β), pro-inflammatory cytokines (TNF-α, IL-1 β, and IL-18), NO and mitochondrial ROS, but a decrease in mitochondrial membrane potential. These results suggest that elevated mitochondrial ROS by LPS/ROT may promote activation of microglia through induction of NLRP3 inflammasome. In addition, this study found that LPS/ROT-treated BV-2 microglial cells displayed a robust induction of both c-Abl phosphorylation on Tyr245 and Tyr412 residues (p-c-Abl), indicators of c-Abl activation, and protein kinase C (PKC)-δ phosphorylation (p-PKC-δ), a downstream mediator of c-Abl activation, while pretreatment with mitochondria-targeted ROS scavengers significantly reduced the levels of p-c-Abl and p-PKC-δ and production of ROS, IL-1β, and IL-18. Interestingly, inhibition of c-Abl by either c-Abl-specific siRNA or pretreatment with dasatinib, a dual kinase inhibitor of both c-Src and c-Abl, reversed the effects of LPS/ROT treatment. Thus, these data suggest that ROT-induced oxidative stress triggers c-Abl activation, leading to exacerbation of LPS-mediated induction of NLRP3 inflammasomes in microglia.

It was also demonstrated that primary microglia stimulated by LPS/ROT exhibited a drastic augmentation of NLRP3 in the mitochondria compared to the control, while dasatinib pretreatment inhibited NLRP3 trafficking to the mitochondria. These data imply that c-Abl inhibition may attenuate ROT-mediated mitochondrial dysfunction, resulting in redistribution of NLRP3 to mitochondria in LPS-primed cells. In addition, this study also showed that LPS/ROT-treated BV-2 cells exhibited a significant increase in NF-κB nuclear translocation and concomitant upregulation of NF-κB-regulated genes including IL-1β, IL-18, and NO, while dasatinib pretreatment markedly attenuated NF-κB activation. These results indicate that the anti-inflammatory effects mediated by dasatinib might, in part, result from inhibition of c-Abl/NF-κB signaling pathways, thereby influencing the NLRP3 inflammasome-mediated pro-inflammatory signaling cascade. Moreover, this study found a sustained and dramatic increase in microtubule-associated proteins 1A/1B light chain 3B, Beclin1, and p62 levels in not only BV-2 cells, but also in primary microglial cells treated with LPS/ROT, compared to that in control cells. However, dasatinib pretreatment and c-Abl siRNA transfection attenuated the increases in these autophagic markers. Furthermore, LPS/ROT-treated BV-2 cells exhibited a marked reduction in lysosomal acidification and transcription factor EB (TFEB) expression levels compared to control cells, while dasatinib pretreatment restored lysosomal acidification and the levels of TFEB expression. These observations suggest that ROT-mediated perturbation of autophagic flux might be further aggravated by LPS. In a neuron-glia co-culture system, it was demonstrated that sequential treatment with LPS/ROT caused a significant increase in p-c-Abl and p-PKC-δ levels and the loss of tyrosine hydroxylase (TH)^+^ cells, while dasatinib restored the loss of TH levels. In addition, dasatinib administration was shown to reduce the expression of NLRP3, mature caspase-1, IL-1β, and ionized calcium-binding adapter molecule-1 in the SN of LPS-induced neuroinflammatory mouse model of PD. Likewise, LPS-induced hyperphosphorylation of c-Abl kinase and PKC-δ as well as induction of autolysosomal markers were ameliorated by dasatinib treatment in the SN of these mice. Lastly, it was demonstrated that sickness behavior as well as motor function of these mice were also improved by dasatinib treatment.

Taken together, this study for the first time suggested that LPS priming and subsequent ROT stimulation induces ROS-dependent aberrant c-Abl activation, thereby exacerbating NLRP3 inflammasome signaling and amplifying the microglial activation response. Indeed, a study showing that microglial expression of IL-1β and IL-18 depends on NLRP3 inflammasomes supports the findings of this study (Gustin et al., 2015). Moreover, a strong correlation between inflammasomes and neurological disorders including AD, PD, and multiple sclerosis (MS) has been reported in many recent studies (Lang et al., 2018). Thus, targeting c-Abl may be an effective therapeutic option for the modulation of inflammasomes and neurotoxicity.

### Spleen Tyrosine Kinase and Janus Kinase 2

Janus kinase 2 (JAK2) belongs to the JAK kinase family comprising four members: JAK1, JAK2, JAK3, and TYK2. It is involved in various cellular signaling activities induced by the activation of diverse receptor families including the type II cytokine receptor family, granulocyte-macrophage colony-stimulating factor receptor family, gp-130 receptor family, and the single chain receptors (Hebenstreit et al., 2005). Spleen tyrosine kinase (SYK), a member of the SYK family comprising SYK and Zeta-chain-associated protein kinase 70, plays a key role in the signaling cascade mediated by immunoreceptors such as B-cell and T-cell receptors (Frommhold et al., 2007; Mócsai et al., 2010). In addition, SYK is known to be recruited to the TLR-associated receptor complex, and thereby contributes to phagocytosis and cytokine expression in various immune cells (Miller et al., 2012). Several previous studies have provided evidence of the involvement of SYK and JAK2 in microglia-mediated inflammatory responses (Combs et al., 1999; Kacimi et al., 2011; Cunningham et al., 2012; Minogue et al., 2012)

A recent study has further indicated the contributing roles of SYK- and JAK-mediated signaling in microglia upon LPS stimulation (Zeng et al., 2014). This study demonstrated that LPS-treated BV-2 cells exhibit an increase in SYK phosphorylation, but pretreatment of DSF-27, a sesquiterpene dimmer compound isolated from *Artemisia argyi*, reversed this increase. In addition, it was found that BV-2 cells treated by either SYK-specific siRNA or DSF-27 pretreatment showed a reduced NO production and phosphorylation of AKT/NF-κB upon LPS stimulation, compared to control cells, suggesting that SYK activation is involved in LPS-mediated AKT/NF-κB activation. In line with these results, DSF-27 pretreatment was shown to inhibit the expression of various inflammatory factors induced by LPS. Considering that both SYK and AKT is potentially involved in the signaling pathways downstream of TREM2, a critical receptor required for DAM activation (Xing et al., 2015; Mecca et al., 2018), future studies should investigate whether both SYK and AKT may contribute to the function of DAM (Mecca et al., 2018).

The study also demonstrated that phosphorylation of JAK2 and signal transducer and activator of transcription 3 (STAT3) was significantly decreased in LPS-treated BV-2 cells by DSF-27 pretreatment. In addition, treatment with AG490, a specific inhibitor of JAK2, was shown to suppress induction of STAT3 and NO in LPS-treated BV-2 cells. Thus, these data suggest that JAK2 exerts its pro-inflammatory effect on microglia after LPS stimulation, and the anti-inflammatory effect of DSF-27 is in part mediated by inhibition of JAK2. It was also shown that DSF-27 treatment suppressed phosphorylation of both extracellular-signal-regulated kinase (ERK) and p38, and that treatment of BV-2 cells with specific inhibitors of these kinases lowered LPS-induced phosphorylation of JAK2 and STAT3 and release of NO. These results imply that both ERK and p38 may function as potential upstream kinases regulating JAK2/STAT3 signaling pathways in microglia. Lastly, in co-culture systems of primary cortical neurons and microglia as well as midbrain neurons and microglia, this study demonstrated that DSF-27 attenuates neuronal degeneration, suggesting a protective role of DSF-27 against microglia-mediated neuronal injury.

Since IL-6 could activate the JAK2/STAT3 signaling pathway, which could induce TNF-α release, resulting in activation of the SYK/NF-κB signaling pathway, both the SYK and JAK2 pathways could promote activation of each other, thereby enhancing the inflammatory responses. Taken together, this study indicates that regulation of the SYK and JAK2/STAT3 signaling pathways could be an effective therapeutic approach for the containment of microglia-mediated neuroinflammation and neurotoxicity (Chiba et al., 2009).

### Serum- and Glucocorticoid-Inducible Kinase 1

Serum- and glucocorticoid-inducible kinase 1 (SGK1) belongs to SGK family comprising three members: SGK1, SGK2, and SGK3. Its expression is regulated by serum and a wide variety of hormones including glucocorticoids (Lang et al., 2006). Although the detailed distribution and function remains unknown, all SGK family members are detected in the CNS (Kobayashi and Cohen, 1999). It has been reported that SGK1 was detected in neurons, astrocytes, oligodendrocytes, and a minor proportion of microglia (Wärntges et al., 2002; Miyata et al., 2011; Slezak et al., 2013). Previous studies have suggested that SGKs play significant roles in neutrophils, Th17 cells, and dendritic cells (Kleinewietfeld et al., 2013; Burgon et al., 2014; Schmid et al., 2014). However, biological functions of SGKs in microglia have been less studied.

Inoue et al. (2016) recently reported that all three members of the SGK family are detected in the mouse cerebral cortex, while SGK1 and SGK3 are detected in several cell lines of microglia including BV-2 and N9. This study revealed that treatment with gsk650394, a pan-SGK inhibitor, significantly inhibited the cellular viability of both BV-2 and N9 cells, suggesting the involvement of basal SGK activity in the maintenance of microglial viability. Pretreatment with gsk650394 further enhanced LPS-mediated translocation of NF-κB and production of pro-inflammatory molecules, including iNOS, NO, and TNF-α in BV-2 cells, and NO production in N9 cells compared to that in untreated control cells. Thus, these results indicate that basal SGK activity is likely associated with the suppression of inflammatory responses of microglial cells.

The same group recently reported the pivotal role of Sgk1 in inhibiting pathological activation of microglia by using Sgk1 KO (Sgk1^-/-^) BV-2 cells, which were created using the clustered regulatory interspaced palindromic repeats/Cas9 system (Asai et al., 2018). This study indicated that functional abolition of Sgk1 in BV-2 cells resulted in amoeboid morphology as well as increased levels of CD68 expression, which are characteristics of activated microglia. Furthermore, LPS-mediated iNOS expression was enhanced in Sgk1^-/-^ BV-2 cells compared with WT BV-2 cells, indicating that disruption of Sgk1 promotes microglial activation. In addition, this study found that Sgk1^-/-^ BV-2 cells proliferate more rapidly than WT BV-2 cells, as demonstrated in a previously study using gsk650394, suggesting that Sgk1 is involved in cellular proliferation. Moreover, compared to that in WT BV-2 cells, Sgk1^-/-^ BV-2 cells exhibited significantly lower AKT phosphorylation and higher ATP susceptibility. Since it has been reported that high doses of ATP repress AKT signaling in microglia and other cells (Bian et al., 2013; Hao et al., 2013), this study assumed that increased susceptibility of Sgk1^-/-^ BV-2 cells to ATP may be at least due to the lower activity of AKT signaling.

Collectively, this study suggests that basal activity of microglial SGK may participate in the inhibition of microglia-mediated neuroinflammation. However, given that SGK1 is implicated in diverse neuronal functions (Lang et al., 2009), preferential targeting of SGK1 in microglia, but not in other cells in the CNS, may clarify the potential *in vivo* role of basal microglial Sgk1 activity in anti-inflammatory responses in the future.

### Dual-Specificity Tyrosine Phosphorylation-Related Kinase 2

The dual-specificity kinases are able to catalyze phosphorylation of not only Ser/Thr residues in their substrates, but also a single Tyr residue in their own activation loop (Becker and Joost, 1999). Dual-specificity tyrosine phosphorylation-related kinase 2 (DYRK2) belongs to the dual-specificity tyrosine phosphorylation-related kinase (DYRK) family comprising five members: DYRK1A, DYRK1B, DYRK2, DYRK3, and DYRK4. It has been previously reported that DYRK2 primes c-Jun and c-Myc phosphorylation in cancer cells and controls p53 phosphorylation on the Ser46 residue upon DNA damage (Yoshida, 2008; Taira et al., 2012; Sun et al., 2017). DYRK2 has also been demonstrated to negatively regulate Type I IFN signaling by promoting TNF receptor associated factor family member-associated NF-κB activator-binding kinase 1 (TBK1) degradation via phosphorylation of TBK1 at the Ser527 residue. Since TBK1 is a downstream molecule of TLR4, which is involved in the activation of NF-κB and the induction of pro-inflammatory cytokine expression (Möser et al., 2015), the potential role of DYRK2 in inflammatory responses has been suggested (An et al., 2015).

A recent study explored the potential role of Dyrk2 in LPS-induced activation of microglia (Xu et al., 2018). This study demonstrated that Dyrk2 is localized in the nucleus, but mainly in the cytoplasm of BV-2 cells. In the course of LPS stimulation for 6 h, this study found that the Dyrk2 level was gradually decreased in the cytoplasm, but increased in the nucleus. The phosphorylation of the NF-κB subunit, p65 (p-p65), remained unchanged in the nucleus, but gradually increased in the cytoplasm for 3 h and later decreased. However, the level of p38 phosphorylation (p-p38) was enhanced in both the cytoplasm and the nucleus. This study also demonstrated that Dyrk2 overexpression in BV-2 cells significantly increased the levels of p-p65, p-AKT, and p-p38 after LPS stimulation, implying that DYRK2 is involved in phosphorylation of these molecules. Interestingly, however, it was found that Dyrk2 overexpression reduced the expression of TNF-α and IL-1β. Considering a previous report demonstrating that AKT activation in microglia is associated with anti-inflammatory effects (Kalantari et al., 2017), it is suggested that decreased production of these cytokines by Dyrk2 overexpression is likely due to AKT activation. Since AKT activation also appears to be modulated by TREM2 or CX3CR1 in microglia (Mecca et al., 2018), a contributing role of DYRK2 in AKT activation mediated by these receptors remains to be further studied in the future. It was also found that Dyrk2 selectively interacted with p38 in the nucleus, while it interacted with AKT, p38, and the NF-κB p65 subunit in the cytoplasm of BV-2 cells.

Although the exact mechanisms underlying the direct or indirect influence of DYRK2 on phosphorylation of these molecules and how DYRK2 is activated or translocated to the nucleus upon LPS stimulation remain to be further investigated, this study implies that DYRK2 is likely involved in phosphorylation of multiple molecules downstream of LPS stimulation, and thereby regulates the production of pro-inflammatory molecules.

### Receptor D’Origine Nantais

Receptor D’Origine Nantais (RON) is a member of Met proto-oncogene family. It is known to be widely expressed in the neurons and microglia, and is activated by macrophage-stimulating protein (MSP), which functions as a neurotrophic factor (Stella et al., 2001; Yu et al., 2016).

A recent study reported that microglial RON is activated upon binding to MSP, resulting in the promotion of the anti-inflammatory process and subsequent tissue repair (Dey et al., 2018). This study demonstrated that the CNS of mice carrying a deletion of ligand binding domain (Ron^-/-^) exhibited an increase in pro-inflammatory molecules including iNOS, COX2, and TNF-α and tissue degradation factors such as MMP2 and MMP9 compared to that of age-matched WT mice. In addition, Ron^-/-^ mice exhibited exacerbated levels of several metabolic markers of CNS stress (phenylalanine, glutamate, hypoxantine, and choline), but decreased levels of healthy neuron markers (*N*-acetyl aspartate and glutamine). These results imply that RON plays protective roles in the CNS. It also indicated that RON expression was detected in CHME-3 cells, a human fetal microglial cell line, in which MSP stimulation resulted in induction of RON expression and a reduction in LPS-mediated production of TNF-α and IL-1β. Using two disease models, this study further demonstrated the involvement of RON in CNS inflammation. In an experimental autoimmune encephalomyelitis mouse model of MS, loss of Ron led to greater disease severity with elevated tissue inflammation in the CNS compared to that in WT mice. Similarly, it has been shown that loss of Ron resulted in exacerbated CNS inflammation with decreased M2 markers and concomitantly increased M1 markers in the diet-induced obesity mice in comparison to control mice (Frommhold et al., 2007). Currently, it is unknown whether RON activation may also be beneficial in other neurodegenerative diseases. However, this study suggests that induction of RON activation selectively in the CNS could potentially alleviate uncontrolled CNS inflammation.

## Epilog

Microglia are essential for proper brain development and maintenance of CNS homeostasis, such as regulation of synaptic maturation, elimination of damaged or dying neurons, and secretion of neurotrophic modulators. However, microglia lose these physiological functions during the course of many CNS diseases and exhibit distinct phenotypic signatures associated with neurodegenerative diseases, in which microglia-mediated neuroinflammation is one of the key pathologic hallmarks (Butovsky and Weiner, 2018). Thus, taming microglia with GPMs toward both maintaining homeostatic function and inhibiting disease-promoting or inflammatory responses could serve the goal of microglial-targeted therapy (Song and Suk, 2017; Suk, 2017; Lee and Suk, 2018).

A recent analysis reported that the likelihood of US FDA approval for CNS drugs is approximately 50% of that for drugs in other areas and that it takes longer for FDA to approve CNS drugs (Schwartz and Deczkowska, 2016). In addition, gradual and more challenging progress has been made in the development of kinase inhibitors for CNS disorders compared to that for other therapeutic areas such as oncology and chronic inflammation, which appears largely due to the intrinsic complexity linked to CNS drug development and partly to the lack of validated druggable targets. However, an increasing number of PKs have emerged as potential drug targets for CNS disorders (Chico et al., 2009; Gunosewoyo et al., 2017; Lee and Suk, 2017), suggesting better opportunities for the development of CNS drugs in the future. However, the lack of relevant biological systems capable of predicting the efficacy and toxicity of treatment has been stated as the most frequent reason for the clinical failure of CNS dug development. Thus, for the success of developing CNS disease-modifying therapies with GPMs including PKs modulators, we should consider the presence of not only species-specific, but also regional brain differences between human and rodent microglia regarding basic biochemistry, gene expression, transcriptomic profiles, and pharmacologic responses (Melief et al., 2012, 2016; Doorn et al., 2015; Bennett et al., 2016; Grabert et al., 2016; Satoh et al., 2016; Gribkoff and Kaczmarek, 2017). Furthermore, given that genes that are regulated during aging are critically different between mouse and human microglia, and that transcriptomic and epigenetic signatures of human microglia are heavily influenced by the environmental conditions (Gosselin et al., 2017). Thus, for the translation of microglial research into clinical practice, it is necessary to investigate whether all the features shown in rodent microglia by certain kinase activities are truly applicable to human microglia by incorporating more physiologically relevant assay systems (Hu et al., 2015).

In this review, we summarized recent studies on the potential role of selected PKs in microglial activation and concomitant induction of neuroinflammation. It will not be surprising to find additional PKs relevant to microglia-mediated neuroinflammation in future studies. Targeting more than one PK target may provide effective approaches for the future interventions of many CNS disorders. Alternatively, drug repositioning with FDA-approved protein kinase inhibitors may facilitate the process of drug development for CNS disorders, such as the recently demonstrated beneficial effect of c-Abl inhibitors in AD and PD (Miller et al., 2012). Since neurotoxicity, another hallmark shared by many neurodegenerative diseases, is either directly affected by neuronal kinases or indirectly by signaling pathways mediated by microglial activation via microglial kinases (Lee and Suk, 2018), targeting common PKs shared by both neuron and microglia may serve as promising therapeutic strategies for neurodegenerative diseases.

In conclusion, a growing number of PKs not only in neurons, but also in non-neuronal cells in the CNS, such as microglia, are emerging as important therapeutic targets and there is rapid advancement and growth in the understanding of microglial function. Therefore, taming microglial activation states by modulating the activity of the PKs described in this review holds great promise for curing or alleviating symptoms associated with many neurodegenerative diseases.

## Author Contributions

S-HL and KS made a substantial intellectual contribution to this work, and wrote the manuscript.

## Conflict of Interest Statement

The authors declare that the research was conducted in the absence of any commercial or financial relationships that could be construed as a potential conflict of interest.
